# Mechanistic Interrogation of Cell Transformation In Vitro: The Transformics Assay as an Exemplar of Oncotransformation

**DOI:** 10.3390/ijms23147603

**Published:** 2022-07-09

**Authors:** Gelsomina Pillo, Maria Grazia Mascolo, Cristina Zanzi, Francesca Rotondo, Stefania Serra, Francesco Bortone, Sandro Grilli, Monica Vaccari, Miriam N. Jacobs, Annamaria Colacci

**Affiliations:** 1 Department of Environmental Health, Agency for Prevention, Environment and Energy, Emilia-Romagna, Viale Filopanti, I-40126 Bologna, Italy; gpillo@arpae.it (G.P.); mmascolo@arpae.it (M.G.M.); czanzi@arpae.it (C.Z.); frotondo@arpae.it (F.R.); stserra@arpae.it (S.S.); monica.vaccari4@unibo.it (M.V.); 2 Department of Experimental, Diagnostic and Specialty Medicine, Section of Cancerology, Alma Mater Studiorum University of Bologna, I-40126 Bologna, Italy; francesco.bortone93@gmail.com (F.B.); sandro.grilli@unibo.it (S.G.); 3 Department of Toxicology, Centre for Radiation, Chemical and Environmental Hazards Public Health England, Chilton OX11 0RQ, UK; miriam.jacobs@phe.gov.uk

**Keywords:** transformics assay, cell transformation assay, transcriptomics, mechanistic understanding, chemical-induced transformation, enrichment analysis, tumor microenvironment, aryl hydrocarbon receptor, in vitro oncotransformation, carcinogenesis

## Abstract

The Transformics Assay is an in vitro test which combines the BALB/c 3T3 Cell Transformation Assay (CTA) with microarray transcriptomics. It has been shown to improve upon the mechanistic understanding of the CTA, helping to identify mechanisms of action leading to chemical-induced transformation thanks to RNA extractions in specific time points along the process of in vitro transformation. In this study, the lowest transforming concentration of the carcinogenic benzo(a)pyrene (B(a)P) has been tested in order to find molecular signatures of initial events relevant for oncotransformation. Application of Enrichment Analysis (Metacore) to the analyses of the results facilitated key biological interpretations. After 72 h of exposure, as a consequence of the molecular initiating event of aryl hydrocarbon receptor (AhR) activation, there is a cascade of cellular events and microenvironment modification, and the immune and inflammatory responses are the main processes involved in cell response. Furthermore, pathways and processes related to cell cycle regulation, cytoskeletal adhesion and remodeling processes, cell differentiation and transformation were observed.

## 1. Introduction

Cancer is a consequence of a complex process requiring the alteration and modification of several biological traits that includes changes at the molecular, cellular, tissue and organ level. In humans, this process takes years to become phenotypically evident and the diagnosis is often made when the organ function is already impaired. 

Addressing this complexity by using appropriate experimental in vitro models that can resemble the biological process is still a challenge.

Cancer development has been associated with chemical exposure since Sir Percival Pott described, in 1775, the occurrence of scrotum cancer in chimney sweepers [1,2]. This first observation paved the way to the discovery of several classes of chemicals acting as carcinogens in human, most (but not all) of which have been demonstrated to act through genotoxic mechanisms, involving a direct damage to DNA [2]. At the beginning of the 21st century, the identification of cancer hallmarks added new perspectives in the understanding of cancer onset and development, following chemical exposure. 

The first steps in the chemical carcinogenesis process regards progressive changes happening at cellular, genetic and epigenetic levels, which can be resumed by the cellular malignant transformation process. In the past 50 years, multidisciplinary approaches in science (with the increasing role of molecular-based approaches) have been applied to identify key alterations that are required for cellular transformation and malignancy and those mechanism through which environmental agents can induce and/or favor such alterations [3,4,5,6,7,8].

Several key molecular events have been recognized such as immortalization, evasion of apoptosis, immune destruction, anti-growth signals dysfunction, uncontrolled growth factors pathways activation, changes in energy metabolism, invasion and degradation of matrix components, inflammation and genome instability. Due to the complexity of events that together contribute to chemical carcinogenesis, it is unlikely that a single method will be able to provide enough information about diverse events/mechanisms of actions and possible interactions [9,10,11]. Particularly, several studies have combined cellular transformation experiments using different cell lines with the transcriptome profiling, in order to explore the malignant transformation process [10,12,13,14,15,16,17,18]. 

Providing insight into carcinogenicity mechanisms is becoming an increasingly appreciated aspect of hazard assessment and is suggested to be the best strategy to drive new developments [7,9]. 

Until relatively recently, the rodent carcinogenesis bioassay (RCB) was regarded as the gold standard to study the carcinogenesis process in vivo, and to identify the carcinogenic properties of both genotoxic and non-genotoxic chemicals. The RCB provides apical endpoints (the tumor in the target organ(s)), some information on the biochemical and morphological alterations accompanying the formation of the tumor, but little or no information on the mechanisms leading to malignancy, or on the key events which mark each step of the process. In contrast, the Cell Transformation Assay (CTA) can be considered to be a tool to model the carcinogenesis process in vitro resembling the multistage process of cell transformation, which can generate relevant mechanistic information. It has been designed to evaluate the effects of chemicals on the growth of specific normal cell types and their potential progression through a transformation process to fully malignant cells. It has been demonstrated that cellular and molecular events involved in the in vitro cell malignant transformation are similar to those of in vivo carcinogenesis. The “focus” which is characterized by spindle-shaped morphology, basophilic staining and changes in cells growth pattern (e.g., multi-layered and anchorage independent growth) is the measurable phenotypic assay end-point.

The CTA was developed in the 1960’s in a variety of different cell lines and protocols, with the intention of replacing the RCB to reduce animal testing, while also providing a less expensive and faster tool to test chemicals for carcinogenicity. During the process to develop the Organization for Economic Cooperation and Development (OECD) test guideline, however, some limitations of the CTA were noted, leading to the decision that the assay could not be used as a stand-alone test for predicting carcinogenesis, but it could be included in an integrated strategy to contribute to the weight-of-evidence for the chemical classification [9,19]. 

Nowadays, the CTAs can be particularly attractive within the field of alternative methods for carcinogenicity due to their potential to detect both genotoxic and non-genotoxic carcinogens. Indeed, mechanistic cancer research has proposed several types of optimization of this assay [11,15,16,17,18]. There are multiple mechanisms of action of non-genotoxic carcinogens, far more so than for genotoxic carcinogens, which induce cancer by interacting directly with DNA and/or the cellular apparatus involved in the preservation of the integrity of the genome [10,20]. Recognized non-genotoxic carcinogenic mechanisms include, and are not limited to, receptor activation, CYP450 induction, stimulation of oxidative stress, chronic cell injury, immune signaling, alterations and interference with intercellular communication [4,21,22].

Recently, the Transformics Assay has been developed to upgrade the BALB/c-3T3 CTA for application to regulatory chemical hazard assessment in addition to mechanistic research [23]. We have previously reported how this method improves the mechanistic understanding of the CTA, by using transcriptomics analysis to facilitate the identification of the key events marking the in vitro cell driven process leading to cell oncotransformation. By using this Transformics Assay approach, we were able to discriminate the adaptive and adverse response of BALB/c 3T3 cells exposed to 3-methylcholantrene (3-MCA), suggesting that for this genotoxic chemical, a threshold may exist [23]. To better understand the events that mark the turning point from the adaptive to the adverse response, here, we report on the testing of the more toxic and carcinogenic chemical, B(a)P. In order to highlight the potential of this method for the description and identification of carcinogens, we have conducted a deeper analysis of genes and pathways involved in the molecular perturbation induced by the chemical.

B(a)P can act via both genotoxic and non-genotoxic Modes of Action [24,25] and is considered the reference chemical for the chemical class of polycyclic aromatic hydrocarbons (PAHs). As such, it is used to identify the carcinogenic potency of other PAHs and of complex mixtures containing PAHs via the AhR [26], one of the receptor mediated molecular initiating events identified for the integrated approach for the testing and assessment of non-genotoxic carcinogen of carcinogens [10]. 

Particularly, B(a)P is a prototypical carcinogen whose toxic effects have been extensively studied, both in laboratory animals and human populations.

B(a)P and other PAHs-induced carcinogenesis instigate a multi-step process, which in the last decade are considered to be initiated and substantially sustained by the reaction with and binding to DNA (oncogenic targets). These events support the genotoxic mode of carcinogenic action. 

Nevertheless, it is now widely recognized that B(a)P can act via non-genotoxic modes of action too, including epigenetic mechanisms involving the AhR signaling pathway, oxidative stress, disruption of mitogenic and other cellular signaling pathways.

The aims of this study are to explore the molecular and cellular events necessary to sustain the transforming activity of B(a)P, at the lowest in vitro concentration capable of sustaining the process of oncotransformation. Use of the Transformics Assay, as reported herein, confirms the ability of the assay to highlight the multiple diverse mechanisms and their plausible interactions leading to cell malignant transformation.

The Transformics Assay is therefore proposed as a suitable method for inclusion within an IATA, particularly relevant for NGTxC, as it provides an in vitro adverse outcome represented and measurable by the “focus”, whose association with particular expressed gene profiles can help to identify mechanisms of action leading to chemical-induced transformation. In this way, the “focus” or ‘foci’ end-point(s), fundamental for the evaluation of the chemical carcinogenic potential, can be supported by direct evidence linking the phenotypic end-point to the molecular events, as demonstrated for two different CTAs [17,23].

## 2. Results

### 2.1. CTA

**Figure 1:**
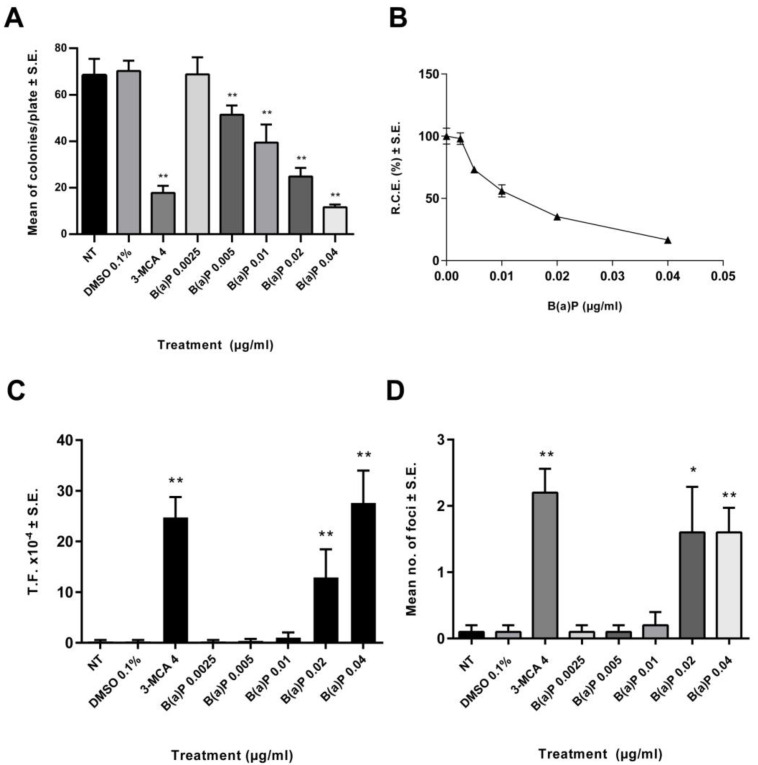
Preliminary CTA results. The test was performed according to the EURL-ECVAM (EU Reference Laboratory European Centre for the Validation of Alternative Methods) validated protocol. Cells were treated 24 h after seeding. Following 72 h exposure, the medium was removed, and the cell cultures were incubated in a humidified incubator at 37 °C with 5% CO_2_. Both cytotoxicity and morphological transformation endpoints have been evaluated by conventional visual scoring. (**A**) Mean number of colonies ± standard error (SE). ** *p* ≤ 0.01 vs. vehicle control, *t*-test. (**B**) Relative Clonal Efficiency (RCE). ** *p* ≤ 0.01 vs. vehicle control, χ^2^-test. (**C**) Transformation frequency (T.F.), calculated on the basis of the foci number and the Absolute Clonal Efficiency (ACE) values ** *p* ≤ 0.01 vs. vehicle control, Poisson rates comparison. (**D**) Mean number of foci ± standard error (SE). * *p* ≤ 0.05 vs. vehicle control, *t*-test. ** *p* ≤ 0.01 vs. vehicle control, *t*-test.

**Figure 2:**
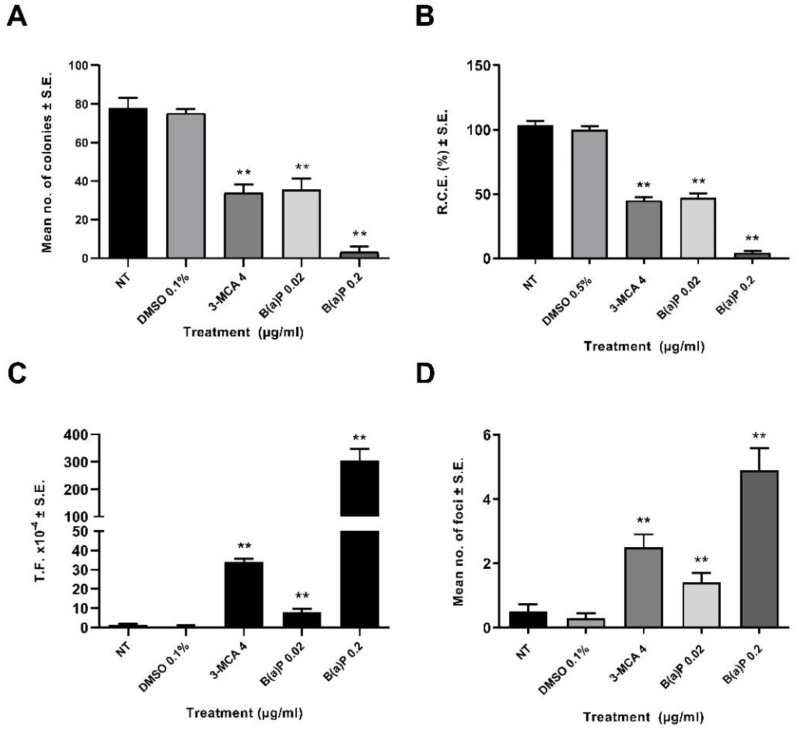
CTA (Transformics Assay) Results. The test was performed according to the EURL-ECVAM validated protocol. Cells were treated 24 h after seeding. Following 72 h exposure, the medium was removed, and the cell cultures were incubated in a humidified incubator at 37 °C with 5% CO_2_. Both cytotoxicity and morphological transformation endpoints have been evaluated by conventional visual scoring. (**A**) Mean number of colonies ± standard error (SE). ** *p* ≤ 0.01 vs. vehicle control, *t*-test. (**B**) Relative Clonal Efficiency (RCE). ** *p* ≤ 0.01 vs. vehicle control, χ²-test. (**C**) Transformation frequency (T.F.), calculated on the basis of the foci number and the Absolute Clonal Efficiency (ACE) values. ** *p* ≤ 0.01 vs. vehicle control, Poisson rates comparison. (**D**) Mean number of foci ± standard error (SE). ** *p* ≤ 0.01 vs. vehicle control, *t*-test.

A preliminary dose range finding CTA was performed (Figure 1). The effects on the cell survival were evaluated on the basis of the cellular capability to form colonies. A statistically significant reduction of cell growth was observed also at low concentrations (Figure 1). The 0.02 µg/mL B(a)P has been identified as the Lowest Transforming Concentration (LTC). This finding has been confirmed in the Transformics Assay CTA (Figure 2) in which Statistically significant cytotoxic and transforming effects were observed (*p* ≤ 0.01) (Figure 2), in accordance to the original requirements of the protocol [27]. 

### 2.2. Statistical Analysis of Microarray Experiment

The *t*-test unpaired (*p* ≤ 0.05, Benjamini–Hochberg) 0.02 μg/mL vs. 0.1% DMSO was performed using GeneSpring software, this indicated an extensive gene perturbation with B(a)P treatment on BALB/c 3T3 cells. Approximately 800 differently expressed genes were identified, among which 459 genes were over-expressed and 347 under-expressed. No fold change cut-off was applied.

### 2.3. Biological Interpretation of Microarray Experiments

The GeneSpring output dataset of 800 significantly modulated genes were analyzed further through the MetaCore V 6.34 and Single Experiment Workflow, which resulted in a total of 577 modulated genes included in the Metacore gene set analysis, corresponding to 667 network objects. Application of MetaCore Enrichment Analysis resulted in several Pathway Maps, Process Networks and Gene Ontology (GO) cellular processes with a statistically significant false discovery rate (FDR ≤ 0.05) (Table 1, Table 2 and Table 3). 

The statistically significant data were quantitatively dominated by the involvement of immune response and inflammatory processes. Additional key pathways and processes that were statistically significant were related to cell cycle regulation (proliferation/apoptosis), cytoskeletal adhesion and remodeling processes, cell differentiation and transformation (Figure S1).

#### 2.3.1. Pathway and Processes Related to Immune Response

**Figure 3:**
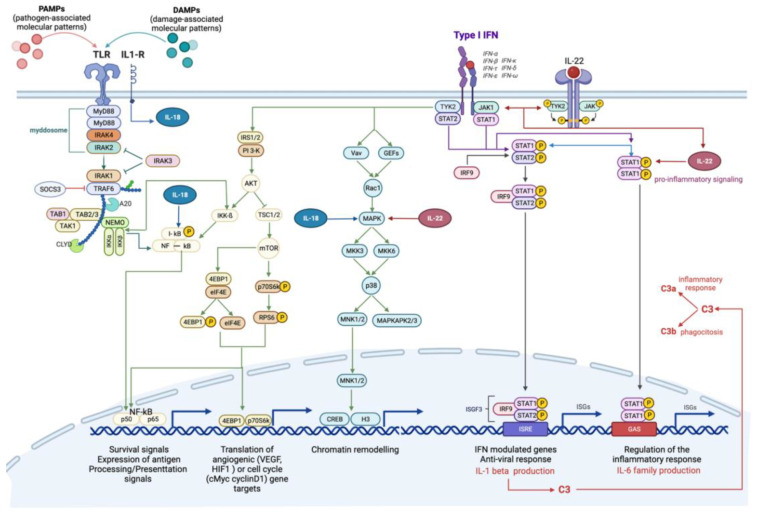
Canonical and non-canonical interferon-mediated signaling pathways. Type 1 interferons (IFN) can trigger signals through the JAK-STAT-mediated canonical pathway and the formation of transcriptional-activator complexes, which translocate into the nucleus and activate the interferon-stimulated response element (ISRE) or IFN-γ-activated site (GAS). The activation of ISRE leads to the transcription of IL-1β, which, in turn, activates the complement cascade. Type 1 IFNs, specifically IFNα, stimulate the activation of IL-22, through IL-22R, which can efficiently stimulate STAT1 and its downstream pathway. IFNs’ non-canonical pathways include IFNα/B signaling via MAPKs pathway, involved in chromatin rearrangement, and IFNα/B signaling via PI3K and NF-kB pathway, involved in survival signals, angiogenesis and cell cycle. The interplay with Toll like receptor (TLR)-mediated pathway is already shown. The activation of T:R by pathogen-associated or damage-associated molecular patterns (PAMPs, DAMPs) triggers the pro-inflammatory NF-kB-mediated signaling pathway, which is inhibited by SOCS3, through the myddosome degradation. Toll-IL1-R receptor activates IL18, which support the activation of NF-kB pathway and interplays with IL-22 to activate MAPK-signaling pathway.

The immune response was characterized by the involvement of several cytokine-dependent pathways, interconnected by common modulated genes (Table 1 and Table 2, Figure 3). Specific pathways of high relevance include: “Immune response_IFN-alpha/beta signaling via JAK/STAT”, “Immune response_IFN-alpha/beta signaling via MAPKs” and “Immune response_IFN-alpha/beta signaling via PI3K and NF-kB pathways”. These pathways are generally activated as a consequence of alpha and beta interferons (IFNs) binding to the respective transmembrane receptors, which, through the activation of intermediate transcription factors such as STAT and IRF, determine inflammatory, immune and proliferative processes (Figure 3). To better address the characterization of single genes, the gene set “Process Network: Inflammation_Interferon signaling” was scanned (Table S1).

The treatment with B(a)P appeared to induce the activation of the complement system, as highlighted by the significant modulation of the related Pathway Maps, in which a considerable over-expression of the C3 gene is shown (Figure 3).

Furthermore, other immune response modulated pathways included interleukin-dependent pathways, associated with pro-inflammatory functions. Of particular interest is the over-expression of the interleukins Il-22 and Il-18 (Table 1; Pathway n. 15; 47) (Figure 3).

Up-regulation of some negative regulators of cytokine signaling and immune responses, which prevent genes from being expressed, were also detected. In particular, the over-expression of Nfkbia, which encodes a member of the NF-kappa-B inhibitor family (I-kb), and Socs3, which encodes the suppressor of cytokine signaling member 3, were highlighted (Figure 3). Both these genes may act in a classic negative feedback loop to regulate cytokine and JAK-STAT signal transduction [28,29].

Process Networks and GO processes analysis also confirmed the importance of the cytokine-mediated inflammation signals (Table 2 and Table 3).

Among the different significantly modulated Process Networks (*p*-values and FDR provided in Table 2), the “Inflammation inflammasome” was of high interest, with the conspicuous presence of INF-related genes in the differential expressed gene list (Table S2). It is also worth noting the Chil1 (Chitinase-like 1, also known as Chi3l1) differential gene expression (FC: +20.65; Table 3: GO processes i.e., n.1-3-5). This gene modulation has been already proposed in other studies as a potential link between the molecular microenvironment related to inflammation and cells transformation [30,31]. Moreover, this gene has been shown to be secreted by Cancer Associated Fibroblasts (CAFs) in breast carcinomas during both early and advanced stages, and to play a central role in tumor promotion, producing a pro-inflammatory and immunosuppressed microenvironment [30,31].

#### 2.3.2. Pathways and Processes Related to EMC Modification and EMT/MET

Several statistically significant pathways and processes were linked to the “Epithelial to Mesenchymal Transition” (EMT), to processes of differentiation and modification of extracellular matrix (ECM) and cells adhesion (Table 1, Table 2 and Table 3). 

From these Pathway Maps analyses, the disruption of cell junctions (i.e., up-regulation of Lef-1, down-stream effector of the WNT signaling), the cytoskeletal remodeling (i.e., down-regulation of Actb, actin beta) and the modification of cell-adhesion and extracellular matrix composition (i.e., up-regulation of Mmp-2 and Mmp-13, Lama 4, Col16a1, Col5a1, Emlin2, Vcam1, down-regulation of Mmp-12, Adamts1 and Adamts5, also known as Aggrecanase-2,) emerged as the main mechanistic outcomes. 

The significance (FDR = 5.307 × 10^−5^) of the Process Network “Signal transduction_WNT signaling” (Table 2; Process Network n. 2; Table S3) and the up-regulation of Wntb5 (FC: +2.037) and Lef-1 (FC: +1.45), together with the down-regulation of Fzd2 (FC: −1.8), support the WNT pathway deregulation. 

In addition, the modulation of “Signal Transduction_TGF-beta, GDF and Activin signaling” and “Signal transduction_NOTCH signaling” are presented in the Process Networks list (Table 2; Process Network n 15;18). 

The down-regulation of Jag1 (Jagged 1, NOTCH ligand), Smad2 and Nanog, crucial transcriptional factors in the TGF-β signaling, were detected, in addition to Edn1 (Endothelin-1), a gene located downstream in this pathway. The up-regulation of the Tgfβr3 gene (Tgf-β type 3 receptor, FC: +1.49) (Process Network n. 15) and Tgf-βi (Tgf-β-induced protein, also known as Beta-ig-h3) (Table 2 Process Network n.3; Table S4) confirmed the TGF-β signaling deregulation [32]. 

Furthermore, the up-regulation of Dcn (Decorin) (Table 2 and Table S4.) is proposed to inhibit the TGF-β.

The down-regulation of Wisp1 and Jag1 could be also evidence of NOTCH1 signaling pathway depression, an event which may occur in sustaining fibroblast proliferation [33,34].

Moreover, the up-regulation of Lif (leukemia inhibitory factor, FC +2.55) (Table 1; pathway n. 40) is a cytokine which belongs to the IL-6 superfamily and has been noted to be an important regulatory factor [35,36,37].

#### 2.3.3. Genes Involved in the Metabolism of Benzo (a) Pyrene

Extensive literature evidence defines B(a)P (also 3-MCA and 2,3,7,8-tetrachlorodibenzo-p-dioxin) as a bifunctional AhR inducer [38,39], which, upon receptor ligand binding and heterodimerization with the heterodimeric transporter protein ARNT, is able to induce both Phase 1 oxidative enzymes (such as the cytochrome P450 enzymes CYP1A1 and CYP1B1) and Phase 2 conjugating enzymes (such as glutathione transferase and UDP-glucuronosyltransferase [39,40,41] 

In this study, a remarkable up-regulation of the Cyp1a1 (FC = +8.17) and Cyp1b1 (FC = +2.76) genes were observed. Positive regulation of the Cyp1a1 and Cyp1b1 genes was also identified in the significantly modulated Process Network “Signal transduction ESR1 nuclear pathway” (Table 2; Process Network n. 25), as was Ugt1a6b over-expression; this gene encodes for UDP-glucuronosyl-transferase (UGT). 

With regard to inducible enzymes, the up-regulation of Ncf4 (Neutrophil cytosolic factor 4, also known as P40phox, FC: +5.070; Table 2; Process Network n. 16) is notable. As a member of the NADPH oxidase subunit, this enzyme plays an important role in NADPH enzyme regulation of oxidase activity and the subsequent production of reactive oxygen species (ROS). A direct transcription regulation of P40phox by AhR has been proposed [42].

## 3. Discussion

Here, we have utilized the “Transformics Assay” to develop an evaluation and analysis of the effect of the lowest transforming concentration of B(a)P using the BALB/c 3T3 A31-1-1 cell line. In this CTA, the 0.02 μg/mL B(a)P concentration resulted in the lowest concentration related to the foci formation. 

The study results reported here consolidate upon those previously reported in the BALB/c 3T3 model validation [43], where the validation report showed an increment in malignant foci formation at the concentration range 0.01–0.02 μg/mL.

The study aimed to identify the molecular and cellular events at the tested threshold concentration following 72 h of exposure. 

A deep perturbation of a large set of immune and inflammatory pathways and processes was observed, showing cross-talk interactions and reciprocal control, essentially ‘immune-editing’. Overall, the data obtained are consistent with principal key events related to the biological process “inflammation”, regardless of the tissue type, specifically (i) cell activation and phenotypic modification, which includes alterations in their secretory activity, activation of biosynthesis pathway, production of pro-inflammation molecules and changes in metabolism and morphology; (ii) increase of molecules with pro-inflammatory action; (iii) recruitment/activation of leukocytes [44] (see Figure S1 for further information about some involved Differential Expressed Genes (DEGs)). 

It is postulated that the first steps in the inflammation signaling could be: mitogen activated kinases (MAPKs) activity and Pattern Recognition Receptors (PPRs) signaling activation [45]. PPRs have been reported to recognize both pathogens (PAMPs, Pathogen-Associated Molecular Patterns) and endogenous ligands released by distressed or damaged cells, generally called DAMPs (Damage-Associated Molecular Patterns) [46,47].

Several modulated pathway are sustained by mitogen-activated protein kinases (MAPKs) activity (Table 1: pathways 1-2-34), which play important roles in the inflammatory response [48].

This result is consistent with other studies which investigated some B(a)P (and other AhR ligands) mechanisms of toxicity mediated by cross-talk between AHR and MAPK [48,49,50].

Many of the inflammation related pathways and processes observed are associated with a “cellular antiviral state”, highlighting overlaps in some sterile and non-sterile stress responses. Indeed, it has been proposed that DNA damage response sensors can mediates the type I interferon (IFN) response and the inflammasome activation [51].

Consistent with this assumption and the known B(a)P genotoxicity, the “Transcription_P53 signaling pathway” (Table 1 pathway n. 39) can be highlighted.

**Figure 4:**
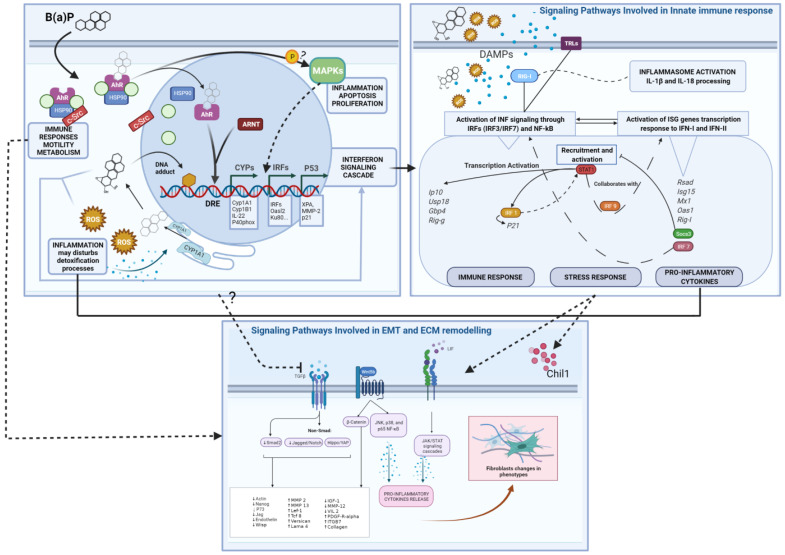
Summary Mode of Action (MoA) diagrams of B(a)P and Differential Expressed Genes. Starting from the left-hand box, the B(a)P-AhR binding and activation as the Molecular Initiating Event leads to Genotoxic and Non-Genotoxic MoAs. Three regulatory factors and transcription programs are highlighted (CYPs, IRFs and P53), leading to the Interferon Signaling Cascade. This leads to DAMPs released from injured cells eliciting an immune response triggered by different pathways, including TLRs and RIG-I. These events enhance the interferons production (type I and probably type III) and subsequent INF signaling response with a positive loop regulation of pro-inflammatory and stress responses (as shown in the right-hand box). Finally, the WNT, LIF-JAK/STAT and TGF-b signaling perturbations and related genes are shown in the box below. Several interactions between different events can be inferred, at the cellular and molecular level, affecting the overall biological response. Abbreviations: Drug Response Element (DRE), Reactive oxygen species (ROS); Damage-Associated Molecular Patterns (DAMPs); Epithelial–Mesenchymal Transition (EMT); Extracellular Matrix (ECM). Created with BioRender.com.

Moreover, in this study, the modulation of the “Immune response_TLR5, TLR7, TLR8 and TLR9 signaling pathways (Toll-like receptors) (Table 1, Pathway n. 42) and the up-regulation of the Rig-I (Retinoic acid-inducible gene 1) together with the modulation of the Process Networks “inflammation inflammasome” and “inflammation interferon signaling” have been observed (Table 2, Tables S1 and S2 and Figure 4). 

The activation of such Pattern Recognition Receptors (RIG-I and TLRs) could represent the initiation step for IRF- and NF-kB-dependent signals activation, leading to the release of type 1 interferons and other cytokines and, consequently, to the JAK/STAT signaling cascade, leading to transactivation of ISG (interferons-stimulated genes) promoters, generating the INF response (Figure 4). 

It is also worth noting the concomitant up-regulation of RIG-I and some ISGs (such as Oas1a), which have been linked to a positive-feedback/feed-forward mechanisms and the formation of an auto-amplification loop RIG-I/IRF [29,49]. Moreover, the over-expression of RIG-I has been connected to the relative inflammasome activation and mature pro-inflammatory interleukins release (such as IL-18) [52,53].

As the results show, several significantly modulated pathways are either directly or indirectly related to the cell transformation process, including EMT-related pathway modulation, ECM molecular signaling remodeling and TGF-b and WNT signaling modulations.

It has been demonstrated that paracrine signaling from CAFs and myofibroblasts can regulate epithelial-mesenchymal plasticity, fibroblast activation, ECM modification and promote tumor progression and fibrosis [18,30,32,35,37,54].

Results from the Enrichment Analysis, as well as the directly observable morphological change in spindle-shaped cells, suggest that fibroblasts are undergoing several modifications, and there are some overlaps with the “CAFs phenotype” [36,37]. Taken in combination, these background signaling events can be considered a composite molecular signal of ECM remodeling, as indicated by the modulation of the relative Pathway maps (Table 1; pathways n 5;10) and Process Networks (Table 2 and Table S4.) Moreover, the scored Lif gene up-regulation has been noted to be associated with a constitutive activation of the JAK1/STAT3 signaling pathway, this sustains the contractile and pro-invasive abilities of fibroblasts [35,36,37]. 

Other gene modulations suggest the inhibition of NOTCH 1 and TGF-β pathways and the deregulation of the WNT signaling pathway (Figure 4). The WNT and TGF-β pathways are both directly and indirectly involved in tissue homeostasis processes of self-renewal and proliferation, differentiation, cell movement and adhesion [55,56,57,58].

The product of Wnt-5b, which is up-regulated in our cell model, is a ligand regulating fibroblasts activation via non-canonical signaling. It occurs in inflammation responses mediating IL-6 and CXCL8 release and JNK, p38 and p65 NF-κB activation [59]. 

While the putative down-regulation of TGF-β signaling has been previously considered controversial, with respect to the classical “molecular phenotype” of myofibroblasts related to CAFs, more recently, Biffi et al. [36] have highlighted differences in CAFs subpopulation in pancreatic ductal adenocarcinoma, discerning between two subtypes characterized by either myofibroblastic or inflammatory phenotypes, with the latter expressing Lif and JAK/STAT activation [36].

While this is a very specific example that so far has not been reported for other cancer types, the fact that this has been observed contributes to an improved weight of evidence for the down-regulation of TGF-β. It is also noteworthy that TGF-β interplay with AhR signaling has been reported [60,61,62].

Interestingly, the LIF/STAT3 and WNT/β-catenin, as well as the inhibition of TGF-β pathways, are also all involved in the maintenance of pluripotency [54]. Considering the final end-point of oncotransformation and not forgetting that EMT can be triggered in a tissue-specific manner, we consider that these combined molecular data illustrate the overall molecular dynamics of the “evolving tumor-microenvironment”.

From this work, we can observe that the AhR-mediated signaling pathway has a major role in triggering the molecular and cellular response to B(a)P in this cell model. The AhR transactivation is supported by the noticeable over-expression of phase I Cyp1a1 and Cyp1b1 genes and of the p40phox enzyme [23,26,42].

The AhR mediates the xenobiotic genotoxicity, but it is also functionally involved in cell adhesion, spreading, migration and modulation of ECM composition, either in physiological cell conditions or following the exposure to xenobiotics, and in particular to B(a)P [41]. Moreover, the AhR is involved in autophagy and EMT processes; these functions can be altered by AhR transmigration and depletion in cytoplasm [63,64].

Our data show the inflammatory/immune response after 72 h of exposure as the main cellular process (Table 1, Table 2 and Table 3 and Figure 4). Results reported in the paper from Mascolo et al. [23] showed a prolonged inflammation state in time. Particularly in this previous study, transforming concentration of 3-MCA treatment had caused the IL-6 production after 28 days from the cellular exposure (end time point of the CTA). Worth noting, the IL-6 cytokines could represent the downstream product of a number of modulated pathways in this B(a)P study. Otherwise, the IL-6 receptor up-regulation can be highlighted (Table 1, Pathway n. 40). It is also worth noting that the inflammation initiation may influence the B(a)P metabolic fate supporting the activation of steps leading to genotoxic damage/injury. This hypothesis is supported by a number of reports showing that exposure to inflammation fostering molecules can slow down the B(a)P metabolism and generate higher levels of B(a)P-DNA adducts [24,65,66,67].

Particularly the phase II enzymes, such as UGTs, are necessary for the transformation of non-polar, exogenous and endogenous compounds into their hydrophilic derivatives. With respect to B(a)P, the activity of UGTs is fundamental for preventing the formation of epoxide BPDE (B(a)P-7, 8-dihydrodiol-9, 10-oxide), a potent mutagenic metabolite [68,69]. Our group has previously noted that the loss in expression of UGTs associated with cell transformation is a crucial event to discriminate the adaptive from the adverse response that leads to transformation [23].

Unlike UGT1A7, UGT1A9 and UGT1A10, UGT1a6 (up-regulated in this study) is unable to detoxify the intermediate metabolite BPD (B(a)P-trans-7,8-dihydrodiol), and thus, is unable to prevent its oxidation and consequent epoxide formation [68,69]. Moreover, down-regulation of some UGTs (but not UGT1A6) have been observed in human hepatocarcinogenesis, while the up-regulation of Ugt1a6 has been observed in the rat hepatocarcinogenesis process [68,69]. Altogether, these data show adverse effects of carcinogens on wide-ranging aspects of the cellular system. Particularly the inflammation signals stimulation, together with oxidative stress effects and inhibition of healing, can build a self-sustaining inflammation system and a transforming microenvironment. At the same time, these immune responses may alter the phase I/phase II metabolism of the carcinogen. Metabolic derived debris (products of oxidative stress and/or unstable metabolites activity) may accumulate in cells and induce the innate immune response to DAMPs, forcing the response to become chronic and maladaptive [21].

According to the multiple roles of the AhR receptor, its transmigration and gene expression activation could be involved in the aberrant immune response [21,23,70], triggering a reactive metabolite cascade that leads to genotoxicity and also promotes fibroblast motility [41] (Figure 4). 

However, further, more specific studies are needed to clarify the complex role of AhR in such processes and eventual ligand-specific effects.

Furthermore, with the identification of a LTC of 0.02 μg/mL B(a)P, we have a preliminary threshold for the CTA when identifying whether a result for an AhR bin of test chemicals is positive or negative, within an IATA. 

Interestingly, in vivo, endpoints predictive of carcinogenicity induced by B(a)P are not the most sensitive toxicity outcomes, and the LTC reported here improves sensitivity for the prediction of oncotransformation, as compared to the RCB. For example, with B(a)P in vivo rodent studies, the critical point of departure of 0.025 mg B(a)P/kg-bw-day for neurotoxicity is used, not the lowest observed adverse level of 0.54 mg B(a)P/kg-bw-day for rodent forestomach carcinogenicity [71].

## 4. Materials and Methods

### 4.1. Experimental Design

**Figure 5:**
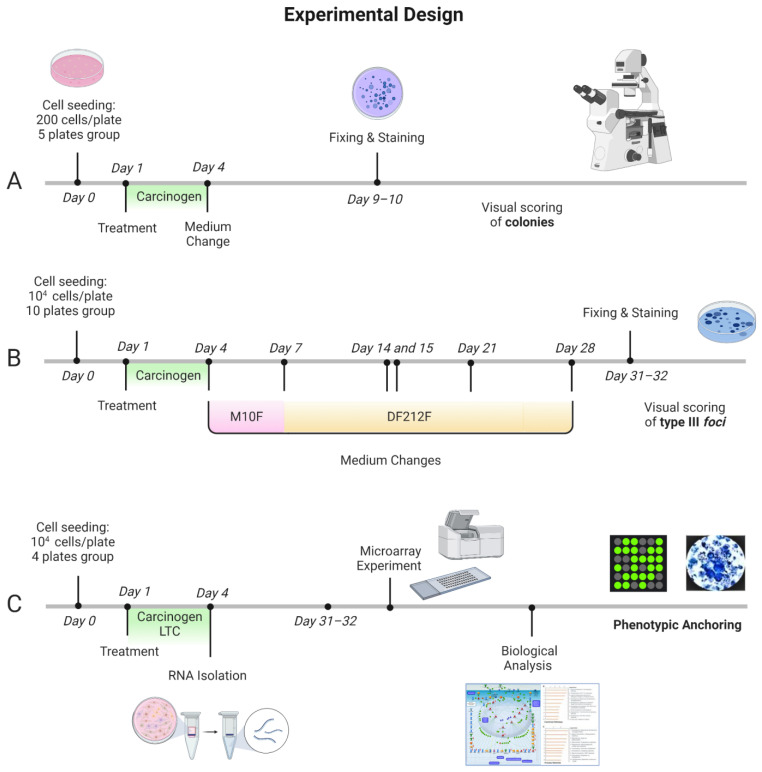
Transformics Assay experimental design. Cell culture experiments were conducted in parallel. A set of plates for each treatment (NT, 0.1% DMSO, 0.02 µg/mL B(a)P, 0.2 µg/mL B(a)P, 4 µg/mL 3-MCA) were maintained in culture for the CTA. Cells were treated 24 h after seeding. Following 72 h exposure, the medium was removed, and the cell cultures were incubated in a humidified incubator at 37 °C with 5% CO_2_. (**A**,**B**). The foci formation served as the phenotypic anchoring for the microarray results. (**A**) Experimental protocol of the Cytotoxicity assay to evaluate the cell survival after the chemical treatment. Five replicates for each test sample were performed. (**B**) Experimental protocol of the Transformation Assay, with medium changes at specified time points. Particularly, M10F medium was used for routine culture: MEM supplemented with 10% Fetal bovine serum FBS and 1% penicillin 10,000 U/mL/streptomycin 10 mg/mL; DF212F medium was used for the late stage of transformation assay: DMEM/F12 with 2 µg/mL insulin, 2% Fetal bovine serum FBS and 1% penicillin 10,000 U/mL/streptomycin 10 mg/mL solution. Ten replicates for each test sample were performed. (**C**) Integrated experimental protocol: Cells were treated 24 h after seeding. Total RNA was isolated after 72 h of exposure. RNA was extracted from cells treated with the Lowest Transforming Concentration (LTC) 0.02 µg/mL B(a)P (16 total plates: 4 technical replicate sand 4 biological replicates) and cells treated with the vehicle 0.1% Dimethyl Sulfoxide (DMSO) (8 total plates: 2 technical replicates and 4 biological replicates) as control. The analysis of the entire transcriptome by microarrays was performed. The microarray experiment was conducted with a glass slide containing 8 × 60 K formatted arrays (Agilent’s Whole Mouse Genome Oligo microarray), four of which were hybridized with the treated cells lysate (four biological replicates), and four with the control lysate (three biological replicates and one technical replicate). The quality control analysis and data normalization were performed by Agilent Feature Extraction. The GeneSpring GX software (Agilent Technologies, Santa Clara, CA, USA) was employed for the statistical analysis. Finally, MetaCore (Thomson Reuters, Toronto, ON, Canana, https://portal.genego.com/, last access 21 December 2021) was used for the biological analysis. Figure created with BioRender.com, last access 25 April 2022.

A preliminary concentration range finding CTA was performed to select the treatment concentrations to be tested in the Transformics Assay. The Transformics Assay experimental design is shown in Figure 5. Cell culture experiments were run in parallel. A set of plates for each treatment (NT, 0.1% DMSO, 0.02 µg/mL B(a)P, 0.2 µg/mL B(a)P, 4 µg/mL 3-MCA) were maintained in culture for the CTA and for the RNA isolation. Cells were treated 24 h after seeding. Total RNA was isolated after 72 h of exposure. RNA was extracted from a set of plates treated with the Lowest Transforming Concentration (LTC) 0.02 µg/mL B(a)P (16 total plates: 4 technical replicate and 4 biological replicates) and a set of plates treated with the vehicle 0.1% Dimethyl Sulfoxide (DMSO) (8 total plates: 2 technical replicates and 4 biological replicates), as control. The analysis of the entire transcriptome by microarrays has been performed. In parallel the CTA experiment was conducted and finalized 31–32 days after seeding, when cells were fixed and stained.

### 4.2. Cells

The original stock of BALB/c 3T3 cells, clone A31-1-1, was obtained from the Health Science Research Resource Bank (Osaka, Japan). Cells were tested, characterized and authenticated by the cell bank for the species origin.

Working cultures were expanded from the original cryopreserved stock. Two vials were thawed at the same time and mixed to create one cell suspension. Cells were grown in M10F, Minimum Essential Medium supplemented with 10% fetal bovine serum (FBS, SigmaAldrich®, St. Louis, MO, USA). Only subconfluent cells were used, and the target cells were not maintained beyond the third passage after thawing. Cultures were maintained in a humidified incubator with an atmosphere of 5% CO_2_ in air at 37 °C.

### 4.3. Chemicals and Solutions

3-Methylcholanthrene (3-MCA, 56-49-5, purity 98.9%), Benzo(a)pyrene (B(a)P, 50-32-8, purity 96%) and Dimethyl Sulfoxide (DMSO, 67-68-5, purity 100%) were obtained from Sigma–Aldrich.

DMSO was used at a 0.1% concentration as the vehicle for the two chemicals and as the negative control.

The stock solution of 3-MCA (4 mg/mL) was delivered to the culture medium to reach the final working concentration of 4 μg/mL. 

The B(a)P working solutions were prepared by diluting sequential stocks solutions in the culture media to obtain concentrations ranging from 0.0025 µg/mL to 0.04 µg/mL, within the preliminary CTA, and the concentration of 0.02 µg/mL in the subsequent Transformics Assay.

### 4.4. CTA and Cytotoxicity Assessment

The test was performed according to the EURL-ECVAM (EU Reference Laboratory European Centre for the Validation of Alternative Methods, European Commission, Joint Research Centre, I-21027 Ispra (VA), Italy) validated protocol [27,72,73]. This protocol includes cytotoxicity testing within the CTA. Details are reported in Supplementary Information, available Online.

A preliminary CTA was performed in order to identify the LTC, by testing B(a)P at concentrations of 0.0025 µg/mL, 0.005 µg/mL, 0.01 µg/mL, 0.02 µg/mL and 0.04 µg/mL (Figure 2). While all the concentrations tested were cytotoxic, they did not all incur transformation. The LTC observed for B(a)P was 0.02 μg/mL, and therefore, this concentration was selected for testing in the CTA for the Transformics Assay. A concentration of 0.2 µg/mL B(a)P was used in the subsequent Transformics CTA, as a positive control.

Cells were treated 24 h after seeding. Following 72 h exposure, the medium was removed, and the cell cultures were incubated in a humidified incubator at 37 °C with 5% CO_2_. The time of exposure was chosen according to EURL-ECVAM validated protocol [27,72,73].

### 4.5. Total RNA Extraction

Total RNA was isolated after 72 h of exposure. RNA was extracted from cells treated with the Lowest Transforming Concentration (LTC) 0.02 µg/mL B(a)P (16 total plates: 4 technical replicate sand 4 biological replicates) and cells treated with the vehicle 0.1% Dimethyl Sulfoxide (DMSO) (8 total plates: 2 technical replicates and 4 biological replicates), as control. Total RNA was isolated by using TRIzol Reagent (Invitrogen, San Diego, CA, USA) followed by purification with Rneasy affinity column (Qiagen, Valencia, CA, USA), according to the manufacturer’s instructions. RNA quality was assessed by Agilent Bioanalyzer 2100 (RNA 6000 Nano LabChip, Agilent Technologies, Palo Alto, CA, USA). Four type 1 biological replicates were obtained for each treatment (0.02 μg/mL B(a)P and 0.1% DMSO). 

### 4.6. Total RNA Labeling and Hybridization

The cRNA was labeled, purified and hybridized on oligonucleotide slides (SurePrint G3 Mouse Gene Expression 8x60K Microarray Kit) using the Low Input Quick Amp Labeling Kit, version 6.9.1, December 2015 (Agilent Technologies, Santa Clara, CA, USA) (www.genomics.agilent.com HYPERLINK http://www.chem.agilent.com/, accessed on 13 May 2018). Four arrays were hybridized with the treated cells lysate (four biological replicates), and four with the control lysate (three biological replicates and one technical replicate).

### 4.7. Statistical Analysis of Microarray Data

Raw data were filtered for intensity and quality signal by using GeneSpring GX (Agilent Technologies). A *t*-test unpaired *p* ≤ 0.05 corrected by Benjamini–Hochberg, was used to select differentially expressed genes after B(a)P treatment in comparison with 0.1% DMSO. No fold change cut-off was applied.

### 4.8. Tools of Biological Interpretation

The list of differentially expressed genes was evaluated by using Metacore™ software V6.34 (Calrivate Analytics), an integrated ‘knowledge-based’ platform with a manually annotated database of protein interactions verified by ‘small experiment’ data and gene–disease associations (https://portal.genego.com/, accessed on 13 May 2018 ). An enrichment analysis was performed using the Analyze Single Experiment workflow, to identify Pathways Maps Process Networks and GO cellular processes altered after 72 h of 0.02 μg/mL B(a)P exposure.

## 5. Conclusions

We have demonstrated how the application of the Transformics method to the CTA can support the elucidation as to how the molecular environment of exposed cells can be resembled, and how it can enable the analysis of several molecular events involved in toxicity and cell transformation. By taking this approach, we have shown how the test method has the ability to highlight diverse mechanisms and their plausible interactions leading to cell malignant transformation. This type of approach could be suitable to enable an interlinked understanding of chemical specific modes of action along the key events of the NGTxC IATA process. 

Some perceived limitations regarding this method can be ascribed to (i) species extrapolation and comparison with the rodent cancer bioassay and (ii) the variability of transcriptomics data and ways in which such data are reported. 

For the former consideration, it is acknowledged that the traditional BALB/c 3T3 CTA showed a concordance of 68%, sensitivity of 75% and specificity of 53%, positive predictivity of 77% and false positive and negative rates of 47 and 25%, respectively, compared to the rodent bioassay [74]. However, it is important to emphasize that the toxicogenomic approach described herein can support the human relevance for data interpretation.

With regard to limitations that may derive from the transcriptomics approach, it is recognized that the transcriptome is of a highly dynamic nature with consequent difficulties in the biological interpretation and that cellular post-transcriptional events are lacking in representation. To overcome this, the Transformics Assay can be performed at any time during the in vitro transformation process, giving the possibility to compare different time-point profiles [23], and furthermore, internationally agreed OECD transcriptomics reporting formats guidance is underway [75]. The latter underlines the wish of the international regulatory community to see greater applications of ‘omics tools for regulatory purposes’, such as that described here.

It is, therefore, proposed that the Transformics Assay can support the use of the CTA to model the carcinogenesis process in vitro within the IATA. Further studies will be conducted to confirm its potential for NGTxC hazard assessment [10].

## Figures and Tables

**Table 1:** The *t*-test unpaired (*p* ≤ 0.05, Benjamini–Hochberg) 0.02 μg/mL treatment vs. vehicle 0.1% DMSO was performed using GeneSpring software. No Fold Change cutoff was applied. In total, 800 significantly modulated genes were analyzed through the MetaCore Single Experiment Workflow. The first 50 statistically significant Pathway Maps in the order of significance are listed (FDR ≤ 0.05). “Gene Ratio” indicates the number of significantly altered genes matching the objects in those specific pathways, out of the total number of genes involved in different specific pathways of the MetaCore database. Finally, up-regulated and down-regulated endpoints indicate individual and/or family genes positively or negatively altered in those specific pathways.

	Enrichment for Pathway Maps					
	Pathway Maps	*p* Value	FDR	Gene Ratio	Up-Regulated Network Objects from Active Data	Down-Regulated Network Objects
1	**Immune response_IFN-alpha/beta signaling via JAK/STAT**	1.419 × 10^−11^	9.964 × 10^−9^	15/64	**IRF1, Mx1, SOCS3, USP18,** **IRF7, p21, IRF9, IP10,** **GBP1, OAS1, ISG15,** **GBP4, RIG-G, RSAD2**	**HIF1A**
2	**Immune response_IFN-alpha/beta signaling via MAPKs**	2.414 × 10^−8^	8.472 × 10^−6^	13/77	**Irgm2, IRF7, Oasl2, p21, IRF9,** **IP10, Oas1g, ISG15,** **PL scramblase 1,** **Ku80, RIG-G, RSAD2**	**JNK (MAPK8-10)**
3	**Transcription_HIF-1 targets**	1.353 × 10^−5^	3.019 × 10^−3^	11/95	**WT1, Angiopoietin 2,** **MMP-2, p21**	**HIF1A, Endothelin-1,** **PGK1, NIP3, GLUT1,** **Nucleophosmin, NANOG**
4	**Development_Regulation of epithelial-to-mesenchymal transition (EMT)**	1.720 × 10^−5^	3.019 × 10^−3^	9/64	**Lef-1, PDGF-R-alpha,** **TCF8,** **MMP-2, WNT**	**Jagged1, Endothelin-1,** **Frizzled, SMAD2**
5	**Cell adhesion_ECM remodeling**	3.966 × 10^−5^	5.305 × 10^−3^	8/55	**Versican, MMP-2, LAMA4,** **MMP-13**	**VIL2 (ezrin),** **Actin cytoskeletal,** **MMP-12, IGF-1**
6	**Development_YAP/TAZ-mediated co-regulation of transcription**	4.534 × 10^−5^	5.305 × 10^−3^	8/56	**Lef-1, Bcl-XL, ID3,**	**HIF1A, Endothelin-1,** **NANOG,** **p73, SMAD2**
7	**Glomerular injury in Lupus Nephritis**	5.815 × 10^−5^	5.831 × 10^−3^	10/92	**IRF1, Decorin, VCAM1,** **CX3CL1,** **RIG-I, IFI56, C3a, IP10**	**HIF1A,** **JNK (MAPK8-10)**
8	**Immune response_IFN-alpha/beta signaling via PI3K and NF-kB pathways**	6.997 × 10^−5^	6.140 × 10^−3^	10/94	**IFIT1, Mx1, IRF7,** **p21, GBP1,** **ISG15, I-kB, RSAD2**	**PI3K cat class IA,** **IRS-1**
9	**Development_TGF-beta-dependent induction of EMT via SMADs**	1.474 × 10^−4^	1.150 × 10^−2^	6/35	**Lef-1,** **TCF8,** **MMP-2**	**Jagged1,** **Endothelin-1,** **SMAD2**
10	**Cell adhesion_Alpha-4 integrins in cell migration and adhesion**	1.734 × 10^−4^	1.187 × 10^−2^	6/36	**VCAM1, ITGB7**	**ERM proteins,** **F-Actin cytoskeleton,** **Actin cytoskeletal,** **PI3K cat class IA**
11	**Signal transduction_Additional pathways of NF-kB activation (in the cytoplasm)**	2.006 × 10^−4^	1.187 × 10^−2^	7/52	**PKC-zeta,** **I-kB, NFKBIA,** **Adenylate cyclase,** **TPL2 (MAP3K8),** **alpha chain CSNK2A1)**	**PI3K cat class IA,** **Casein kinase II**
12	**Development_Growth factors in regulation of oligodendrocyte precursor cell survival**	2.030 × 10^−4^	1.187 × 10^−2^	6/37	**PDGF-R-alpha,** **Bcl-XL,** **NFKBIA**	**PI3K cat class IA,** **IRS-1,** **IGF-1**
13	**Immune response_Classical complement pathway**	2.266 × 10^−4^	1.223 × 10^−2^	7/53	**C3c, C3a, C3b,** **C3dg, C3, iC3b, C1s**	
14	**Apoptosis and survival_APRIL and BAFF signaling**	2.739 × 10^−4^	1.373 × 10^−2^	6/39	**NF-AT2 (NFATC1),** **Bcl-XL, I-kB,** **RelB (NF-kB subunit)**	**JNK (MAPK8-10),** **TACI (TNFRSF13B)**
15	**Development_The role of GDNF ligand family/RET receptor in cell survival, growth and proliferation**	3.062 × 10^−4^	1.386 × 10^−2^	9/92	**GFRalpha2,** **Bcl-XL,** **NFKBIA,** **IL-22**	**F-Actin cytoskeleton,** **PI3K cat class IA,** **JNK2 (MAPK9),** **HIF1A, IRS-1**
16	**Development_Role of Activin A in cell differentiation and proliferation**	3.159 × 10^−4^	1.386 × 10^−2^	6/40	**Adenylate cyclase,** **p21,** **MAD, LHX3**	**NANOG,** **SMAD2**
17	**Immune response_TNF-R2 signaling pathways**	6.077 × 10^−4^	2.396 × 10^−2^	6/45	**TRAF1, Bcl-XL,** **I-kB, BMF**	**PI3K cat class IA,** **JNK (MAPK8-10)**
18	**Signal transduction_Additional pathways of NF-kB activation (in the nucleus)**	6.143 × 10^−4^	2.396 × 10^−2^	5/30	**PKC-zeta, STO,** **Adenylate cyclase,** **I-kB, NFKBIA**	
19	**Immune response_IL-1 signaling pathway**	6.724 × 10^−4^	2.405 × 10^−2^	8/82	**IRF1, PKC-zeta, IP10,** **TPL2 (MAP3K8),** **I-kB, MMP-13**	**PI3K cat class IA,** **JNK (MAPK8-10)**
20	**Transcription_Androgen Receptor nuclear signaling**	6.853 × 10^−4^	2.405 × 10^−2^	6/46	**MMP-2,** **p21, WNT**	**VIL2 (ezrin),** **Frizzled, IGF-1**
21	**Immune response_Histamine H1 receptor signaling in immune response**	7.703 × 10^−4^	2.458 × 10^−2^	6/47	**NF-AT2(NFATC1), VCAM1,** **I-kB, NFKBIA,** **MMP-13**	**JNK(MAPK8-10)**
22	**Development_PIP3 signaling in cardiac myocytes**	7.703 × 10^−4^	2.458 × 10^−2^	6/47	**PKC-zeta, Bcl-XL**	**G-protein alpha-12 family,** **PI3K cat class IA,** **IRS-1, IGF-1**
23	**Muscle contraction_Relaxin signaling pathway**	8.632 × 10^−4^	2.635 × 10^−2^	6/48	**PKC-zeta, MMP-2,** **I-kB, NFKBIA**	**PI3K cat class IA (p110-beta), Endothelin-1**
24	**Ligand-independent activation of Androgen receptor in Prostate Cancer**	9.668 × 10^−4^	2.795 × 10^−2^	7/67	**Tcf (Lef), Bcl-XL**	**PP2A regulatory,** **Frizzled,** **PI3K cat class IA,** **IRS-1, IGF-1**
25	**Immune response_Lectin induced complement pathway**	1.075 × 10^−3^	2.795 × 10^−2^	6/50	**C3c, C3a, C3b,** **C3dg, C3, iC3b**	
26	**G-protein signaling_RhoA regulation pathway**	1.111 × 10^−3^	2.795 × 10^−2^	5/34	**ARHGEF3**	**VIL2 (ezrin), PRK1,** **G-protein alpha-12 family, IGF-1**
27	**Androgen receptor activation and downstream signaling in Prostate cancer**	1.135 × 10^−3^	2.795 × 10^−2^	9/110	**ER81, Bcl-XL,** **p21, IL6RA**	**VIL2 (ezrin), Versican,** **PI3K cat class IA (p110-beta),** **IRS-1, IGF-1**
28	**Signal transduction_PKA signaling**	1.195 × 10^−3^	2.795 × 10^−2^	6/51	**LBC, Adenylate cyclase,** **NFKBIA**	**PP2A regulatory,** **AKAP12,** **G-protein alpha-12 family**
29	**Development_IGF-1 receptor signaling**	1.195 × 10^−3^	2.795 × 10^−2^	6/51	**PKC-zeta, Bcl-XL,** **I-kB**	**PI3K cat class IA,** **IRS-1, IGF-1**
30	**Some pathways of EMT in cancer cells**	1.195 × 10^−3^	2.795 × 10^−2^	6/51	**Lef-1, PDGF receptor,** **PDGF-R-alpha, I-kB**	**PI3K cat class IA,** **Endothelin-1**
31	**Immune response_IL-9 signaling pathway**	1.449 × 10^−3^	3.115 × 10^−2^	5/36	**SOCS3, I-kB,** **IL-22**	**PI3K cat class IA,** **IRS-1**
32	**Apoptosis and survival_Role of PKR in stress-induced apoptosis**	1.464 × 10^−3^	3.115 × 10^−2^	6/53	**IRF1, p21,** **I-kB, NFKBIA**	**PACT,** **PP2A regulatory**
33	**Immune response_Alternative complement pathway**	1.464 × 10^−3^	3.115 × 10^−2^	6/53	**C3c, C3a, C3b,** **C3dg, C3, iC3b**	
34	**Immune response_IL-3 signaling via JAK/STAT, p38, JNK and NF-kB**	1.540 × 10^−3^	3.179 × 10^−2^	8/93	**SOCS3,** **Spi2a,** **Bcl-XL, I-kB**	**PI3K cat class IA,** **DHA2, MKP-1,** **TACI (TNFRSF13B)**
35	**Immune response_Platelet activating factor/PTAFR pathway signaling**	1.778 × 10^−3^	3.374 × 10^−2^	6/55	**NF-AT,** **NF-AT2 (NFATC1),** **Adenylate cyclase,** **NFKBIA**	**F-Actin cytoskeleton,** **PI3K cat class IA**
36	**Immune response_CD28 signaling**	1.778 × 10^−3^	3.374 × 10^−2^	6/55	**NF-AT,** **NF-AT2 (NFATC1),** **Bcl-XL, I-kB**	**PI3K cat class IA,** **JNK (MAPK8-10)**
37	**PGE2 pathways in cancer**	1.778 × 10^−3^	3.374 × 10^−2^	6/55	**Tcf (Lef), Lef-1,** **Adenylate cyclase,** **COX-1 (PTGS1)**	**HIF1A,** **PI3K cat class IA (p110-beta)**
38	**G-protein signaling_G-Protein alpha-12 signaling pathway**	1.856 × 10^−3^	3.429 × 10^−2^	5/38	**LBC**	**G-protein alpha-12 family,** **PI3K cat class IA,** **JNK (MAPK8-10), M-Ras**
39	**Transcription_P53 signaling pathway**	2.089 × 10^−3^	3.578 × 10^−2^	5/39	**XPA,** **MMP-2, p21**	**JNK (MAPK8-10),** **MKP-1**
40	**Development_Cytokine-mediated regulation of megakaryopoiesis**	2.141 × 10^−3^	3.578 × 10^−2^	6/57	**IRF1, Bcl-XL,** **p21, LIF, sIL6-RA**	**PI3K cat class IA**
41	**Immune response_Role of PKR in stress-induced antiviral cell response**	2.141 × 10^−3^	3.578 × 10^−2^	6/57	**IRF1, IRF7,** **I-kB, NFKBIA**	**PACT,** **JNK (MAPK8-10)**
42	**Immune response_TLR5, TLR7, TLR8 and TLR9 signaling pathways**	2.141 × 10^−3^	3.578 × 10^−2^	6/57	**IRF1, IRF7, I-kB,** **TPL2 (MAP3K8)**	**PI3K cat class IA,** **JNK (MAPK8-10)**
43	**Development_Prolactin receptor signaling**	2.341 × 10^−3^	3.737 × 10^−2^	6/58	**IRF1, SOCS3,** **Bcl-XL, OAS1**	**PI3K cat class IA,** **IRS-1**
44	**Apoptosis and survival_NGF activation of NF-kB**	2.342 × 10^−3^	3.737 × 10^−2^	5/40	**PKC-zeta, Bcl-XL,** **I-kB, NFKBIA**	**PI3K cat class IA**
45	**Main growth factor signaling cascades in multiple myeloma cells**	2.617 × 10^−3^	3.994 × 10^−2^	5/41	**I-kB**	**HIF1A,** **PI3K cat class IA,** **IRS-1, IGF-1**
46	**Development_Regulation of lung epithelial progenitor cell differentiation**	2.617 × 10^−3^	3.994 × 10^−2^	5/41	**Tcf (Lef),** **WNT**	**Jagged1, FZD2,** **Frizzled,**
47	**Immune response_IL-18 signaling**	2.785 × 10^−3^	4.074 × 10^−2^	6/60	**VCAM1, IL-18,** **Bcl-XS, I-kB**	**PI3K cat class IA,** **JNK (MAPK8-10)**
48	**Immune response_LTBR1 signaling**	2.785 × 10^−3^	4.074 × 10^−2^	6/60	**PKC, I-kB** **Adenylate cyclase,**	**PI3K cat class IA,** **JNK (MAPK8-10), YES**
49	**Apoptosis and survival_Anti-apoptotic TNFs/NF-kB/Bcl-2 pathway**	2.915 × 10^−3^	4.176 × 10^−2^	5/42	**PKC-zeta, Bcl-XL,** **I-kB,** **RelB (NF-kB subunit)**	**TACI (TNFRSF13B)**
50	**Signal transduction_AKT signaling**	3.236 × 10^−3^	4.544E × 10^−2^	5/43	**Bcl-XL, p21,** **I-kB**	**PI3K cat class IA,** **IRS-1**

**Table 2:** The *t*−test unpaired (*p* ≤ 0.05, Benjamini–Hochberg) 0.02 μg/mL treatment vs. vehicle 0.1% DMSO was performed using GeneSpring software. No Fold Change cutoff was applied. 800 significantly modulated genes were analyzed through the MetaCore Single Experiment Workflow. Statistically significant Process Networks in the order of significance are listed (FDR ≤ 0.05). “Gene Ratio” indicates the number of significantly altered genes matching the endpoints in those specific Process Networks, out of the total number of genes involved in different specific Networks of the MetaCore database. Finally, Network Objects from active Data indicate individual and/or family genes altered in those specific Process Network.

	Enrichment for Process Networks				
	Process Networks	*p*-Value	FDR	Gene Ratio	Network Objects from Active Data
1	**Immune response_Phagocytosis**	6.475 × 10^−7^	5.307 × 10^−5^	24/223	MANR, ERM proteins, VIL2 (ezrin), Actin cytoskeletal, Actin, PI3K cat class IA, PI3K cat class IA (p110-beta), JNK (MAPK8-10), JNK2 (MAPK9), MRLC, C3b, C3dg, C3, iC3b, p40-phox, ENDO180, BLNK, HDL proteins, Casein kinase II, alpha chain (CSNK2A1), Casein kinase II, alpha chains, I-kB, NFKBIA, Casein kinase II, alpha’ chain (CSNK2A2), C/EBP
2	**Signal transduction_WNT signaling**	6.760 × 10^−7^	5.307 × 10^−5^	21/177	PP2A regulatory, NF-AT, NF-AT2 (NFATC1), Tcf (Lef), Lef-1, AKAP12, FZD2, Frizzled, JNK (MAPK8-10), JNK2 (MAPK9), MMP-2, WISP1, Adenylate cyclase, NANOG, p73, Casein kinase II, alpha chain (CSNK2A1), Casein kinase II, alpha chains, Casein kinase II, WNT5B, WNT, Casein kinase II, alpha’ chain (CSNK2A2)
3	**Cell adhesion_Cell-matrix interactions**	3.832 × 10^−5^	2.006 × 10^−3^	20/211	Perlecan, ADAM23, ECM1, Aggrecanase-2, Versican, Decorin, COL16A1, BETA-IG-H3, COL5A1, Collagen V, MMP-12, MMP-2, WISP1, Connexin 43, LAMA4, EMILIN-2, ADAM-TS1, ITGB7, TSG-6, MMP-13
4	**Immune response_BCR pathway**	7.205 × 10^−5^	2.494 × 10^−3^	15/137	NF-AT, NF-AT2 (NFATC1), Actin cytoskeletal, Actin, PI3K cat class IA, PI3K cat class IA (p110-beta), JNK (MAPK8-10), JNK2 (MAPK9), C3dg, SHIP, Bcl-XL, BLNK, Casein kinase II, alpha chain (CSNK2A1), I-kB, NFKBIA
5	**Inflammation_IFN-gamma signaling**	9.114 × 10^−5^	2.494 × 10^−3^	13/109	PACT, IRF1, IFI16, IL-18, PI3K cat class IA, PI3K cat class IA (p110-beta), Bcl-XL, p21, IRF9, IP10, EIF2S3, I-kB, NFKBIA
6	**Immune response_Phagosome in antigen presentation**	9.531 × 10^−5^	2.494 × 10^−3^	21/243	ERM proteins, VIL2 (ezrin), Actin cytoskeletal, Actin, PI3K cat class IA, PI3K cat class IA (p110-beta), JNK (MAPK8-10), JNK2 (MAPK9), PSMB9, C3dg, C3, iC3b, SEC15L, PSME3, ENDO180, BLNK, Casein kinase II, alpha chain (CSNK2A1), Casein kinase II, alpha chains, I-kB, NFKBIA, Casein kinase II, alpha’ chain (CSNK2A2)
7	**Inflammation_Interferon signaling**	3.945 × 10^−4^	8.848 × 10^−3^	12/110	IRF1, SOCS3, IFI44, CCL8, IRF7, IFI56, MxA, Bcl-XL, IRF9, GBP1, GBP2, ISG15
8	**Cell adhesion_Cadherins**	5.422 × 10^−4^	1.064 × 10^−2^	16/182	Tcf (Lef), PKC, Actin cytoskeletal, Actin, Frizzled, G-protein alpha-12, PI3K cat class IA, WISP1, MAST205, Casein kinase II, alpha chain (CSNK2A1), Casein kinase II, alpha chains, Casein kinase II, ACP1, WNT, Casein kinase II, alpha’ chain (CSNK2A2), YES
9	**Development_Regulation of angiogenesis**	6.553 × 10^−4^	1.143 × 10^−2^	18/222	HIF1A, PKC, PRK1, WT1, IL-18, TCF8, PI3K cat class IA, FXR, Endothelin-1, MMP-2, Cathepsin B, Connexin 43, Syndecan-3, Angiogenin, p21, SMAD2, I-kB, RelB (NF-kB subunit)
10	**Inflammation_IL-6 signaling**	8.080 × 10^−4^	1.269 × 10^−2^	12/119	SOCS3, PI3K cat class IA, PI3K cat class IA (p110-beta), SAA3, C3, p21, HDL proteins, I-kB, NFKBIA, C/EBP, IL-22, Alpha1-globin
11	**Inflammation_MIF signaling**	1.079 × 10^−3^	1.500 × 10^−2^	13/140	PLA2, PKC, VCAM1, JNK (MAPK8-10), JNK2 (MAPK9), Adenylate cyclase type VII, Adenylate cyclase, Casein kinase II, alpha chain (CSNK2A1), Casein kinase II, alpha chains, Casein kinase II, I-kB, NFKBIA, Casein kinase II, alpha’ chain (CSNK2A2)
12	**Cell cycle_G1-S Growth factor regulation**	1.146 × 10^−3^	1.500 × 10^−2^	16/195	PP2A regulatory, IRF1, Tcf (Lef), PKC, PDGF-R-alpha, PI3K cat class IA, PI3K cat class IA (p110-beta), JNK (MAPK8-10), JNK2 (MAPK9), p21, MAD, IRS-1, I-kB, NFKBIA, RelB (NF-kB subunit), IGF-1
13	**Cell adhesion_Cell junctions**	1.413 × 10^−3^	1.707 × 10^−2^	14/162	ATP1B1, Tcf (Lef), PKC-zeta, PKC, Actin cytoskeletal, Actin, PI3K cat class IA, Endothelin-1, Connexin 43, Casein kinase II, alpha chain (CSNK2A1), Casein kinase II, Casein kinase II alpha chain (CSNK2A2), YES
14	**Inflammation_Amphoterin signaling**	2.500 × 10^−3^	2.678 × 10^−2^	11/118	VCAM1, Actin cytoskeletal, Actin, PI3K cat class IA, PI3K cat class IA (p110-beta), JNK (MAPK8-10), JNK2 (MAPK9), MRLC, I-kB, NFKBIA, MMP-13
15	**Signal Transduction_TGF-beta, GDF and Activin signaling**	2.559 × 10^−3^	2.678 × 10^−2^	13/154	PP2A regulatory, HIF1A, Lef-1, PKC, PI3K cat class IA, JNK (MAPK8-10), IRF7, UNRIP, p21, SMAD2, IRS-1, IGF-1, TGF-beta receptor type III (betaglycan)
16	**Inflammation_Histamine signaling**	2.852 × 10^−3^	2.752 × 10^−2^	16/213	PLA2, NF-AT2 (NFATC1), VCAM1, Actin cytoskeletal, Actin, IL-18, JNK(MAPK8-10), JNK2 (MAPK9), MDP1, p40-phox, Adenylate cyclase type VII, Adenylate cyclase, IP10, COX-1 (PTGS1), I-kB, NFKBIA
17	**Inflammation_IL-10 anti-inflammatory response**	3.016 × 10^−3^	2.752 × 10^−2^	9/87	SOCS3, Nucleolysin TIAR, PI3K cat class IA, PI3K cat class IA (p110-beta), MMP-2, Bcl-XL, I-kB, NFKBIA, MMP-13
18	**Signal transduction_NOTCH signaling**	3.155 × 10^−3^	2.752 × 10^−2^	17/235	Jagged1, HIF1A, PDGF receptor, PDGF-R-alpha, FZD2, Frizzled, PI3K cat class IA, JNK (MAPK8-10), JNK2 (MAPK9), GLUT1, p21, p73, SMAD2, WNT5B, WNT, I-kB, NFKBIA
19	**Inflammation_Innate inflammatory response**	3.772 × 10^−3^	3.117 × 10^−2^	14/180	PLA2, PKC-zeta, IL-18, JNK(MAPK8-10), JNK2 (MAPK9), IRF7, C3a, C3b, C3, PGRP-S, IP10, COX-1 (PTGS1), I-kB, NFKBIA
20	**Reproduction_Male sex differentiation**	4.441 × 10^−3^	3.487 × 10^−2^	17/243	PAFAH alpha (LIS1), PKC-zeta, PKC, PMEPA1, PDGF receptor, PDGF-R-alpha, WT1, BAPX1, AMH type II receptor, Adenylate cyclase type VII, p21, NANOG, STK39, Olfactory receptor, Casein kinase II, Casein kinase II, alpha’ chain (CSNK2A2), IGF-1
21	**Development_EMT_Regulation of epithelial-to-mesenchymal transition**	4.682 × 10^−3^	3.501 × 10^−2^	16/224	Jagged1, HIF1A, NF-AT2 (NFATC1), Lef-1, Actin, PDGF receptor, PDGF-R-alpha, Frizzled, TCF8, PI3K cat class IA, JNK (MAPK8-10), Endothelin-1, MMP-2, SMAD2, WNT, I-kB
22	**Immune response_TCR signaling**	7.223 × 10^−3^	4.933 × 10^−2^	13/174	NF-AT, NF-AT2 (NFATC1), CD137(TNFRSF9), Actin cytoskeletal, Actin, PI3K cat class IA, JNK (MAPK8-10), JNK2 (MAPK9), TRAF1, Bcl-XL, TPL2 (MAP3K8), I-kB, NFKBIA
23	**Inflammation_Inflammasome**	7.597 × 10^−3^	4.933 × 10^−2^	10/118	PACT, RIG-I, IL-18, JNK (MAPK8-10), IRF7, TXNIP (VDUP1), EIF2S3, ISG15, I-kB, NFKBIA
24	**Inflammation_IgE signaling**	7.782 × 10^−3^	4.933 × 10^−2^	11/137	PLA2, NF-AT, NF-AT2 (NFATC1), PI3K cat class IA, PI3K cat class IA (p110-beta), JNK (MAPK8-10), JNK2 (MAPK9), MDP1, BLNK, I-kB, NFKBIA
25	**Signal transduction_ESR1-nuclear pathway**	7.855 × 10^−3^	4.933 × 10^−2^	15/216	SOCS3, WT1, PI3K cat class IA, MPG, SET, LBC, C3, Adenylate cyclase type VII, Adenylate cyclase, RAMP3, CYP1A1, p21, CYP1B1, IRS-1, IGF-1

**Table 3:** Significantly modulated genes resulted from GeneSpring analysis were analyzed further through the MetaCore Single Experiment Workflow. The first 10 statistically significant Gene Ontology (GO) cellular processes are listed (FDR ≤ 0.05). “Gene Ratio” indicates the number of significantly altered genes matching the objects in those specific GO Process out of the total number of genes involved.

	GO Process	*p* Value	FDR	Gene Ratio
1	**Response to stress**	3.713 × 10^−22^	2.771 × 10^−18^	**233/4937**
2	**Cellular response to cytokine stimulus**	3.510 × 10^−19^	1.310 × 10^−15^	**98/1398**
3	**Response to cytokine**	2.914 × 10^−18^	7.248 × 10^−15^	**105/1611**
4	**Response to external stimulus**	1.159 × 10^−17^	2.163 × 10^−14^	**159/3089**
5	**Cellular response to organic substance**	4.898 × 10^−17^	6.115 × 10^−14^	**170/3457**
6	**Response to organic substance**	4.917 × 10^−17^	6.115 × 10^−14^	**207/4579**
7	**Cellular response to chemical stimulus**	4.374 × 10^−16^	4.663 × 10^−13^	**190/4141**
8	**Negative regulation of biological process**	7.285 × 10^−15^	6.795 × 10^−12^	**269/6845**
9	**Response to interferon-beta**	5.701 × 10^−14^	4.727 × 10^−11^	**17/57**
10	**Response to virus**	1.456 × 10^−13^	1.087 × 10^−10^	**41/403**

## Data Availability

Microarray experimental design and protocols, together with the complete raw data-set, are available in the EBI microarray data public repository Arrayexpress http://www.ebi.ac.uk/arrayexpress/ (accessed on 29 April 2021), accession number E-MTAB-10405.

## Supplementary Materials

The following supporting information can be downloaded at: https://www.mdpi.com/article/10.3390/ijms23147603/s1.
