# Supplementary material for: Mechanistic Interrogation of Cell Transformation In Vitro: The Transformics Assay as an Exemplar of Oncotransformation
Source: Int J Mol Sci. 2022 Jul 9;23(14):7603. doi: 10.3390/ijms23147603 (PMC9321586; doi:10.3390/ijms23147603)
Supplement: Supplementary file 1 [file ijms-23-07603-s001.zip › ijms-1692352-supplementary.pdf]

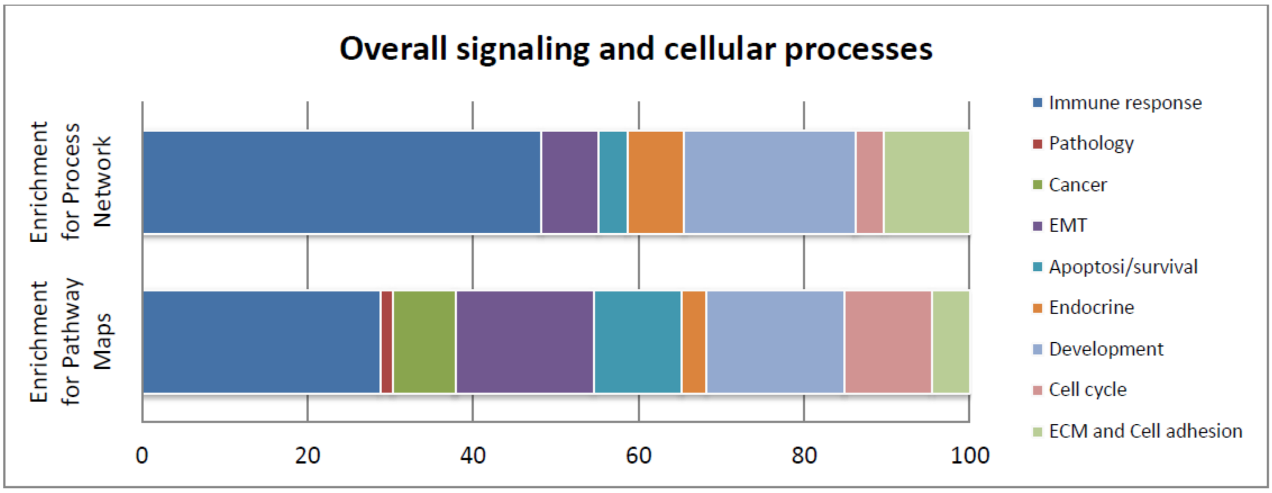

Figure S1. Relative distribution of signalings and cellular processes based on results from the Metacore Enrichment Analysis.

Table S1. DEGs List of the Process Network "Inflammation_Interferon signaling" (Table 2); pValue: 3.945×10^{-4}; FDR: 8.848×10^{-3}; Gene Ratio: 12/110

GENE	DESCRIPTION	FC
**Bcl-XL**	BCL2 like 1, apoptosis regulator during development and tissue homeostasis. Alternative splicing results in multiple transcript variants	**1.120**
**CCL8**	C-C motif chemokine ligand 8, cytokine that displays chemotactic activity for monocytes, concurring in immunoregulatory and inflammatory processes.	**2.437**
**GBP1**	interferon-induced guanylate-binding protein 1, exhibits antiviral activity and broad host protection against different pathogen classes.	**2.278**
**GBP2**	interferon-induced guanylate-binding protein 2, exhibits antiviral activity and broad host protection against different pathogen classes.	**2.467**
**IFI44**	interferon-induced protein 44, protein that aggregates to form microtubular structures.	**2.461**
**IFI56**	interferon induced protein with tetratricopeptide repeats 1B like 2, antiviral RNA-binding protein. The encoded protein may inhibit viral replication and translational initiation.	**2.920**
**IRF1**	interferon regulatory factor 1, transcriptional regulator concurring in several cellular responses. These include the regulation of IFN and IFN-inducible genes, host response to viral and bacterial infections, regulation of many genes expressed during hematopoiesis, inflammation, immune responses and cell proliferation and differentiation, regulation of the cell cycle and induction of growth arrest and programmed cell death following DNA damage.	**1.532**
**IRF7**	Interferon regulatory factor 7, key transcriptional regulator of type I interferon (IFN)-dependent immune responses. Regulates the transcription of type I IFN genes (IFN-alpha and IFN-beta) and IFN-stimulated genes (ISG) by binding to an interferon-stimulated response element (ISRE) in their promoters. Required during both the early and late phases of the IFN gene induction but is more critical for the late than for the early phase.	**3.640**
**IRF9**	Interferon regulatory factor 9, transcription factor that mediates signaling by type I IFNs (IFN-alpha and IFN-beta).	**1.647**
**ISG15**	ISG15 ubiquitin-like modifier, Ubiquitin-like protein which plays a key role in the innate immune response to viral infection.	**1.822**
**MxA**	MX dynamin-like GTPase 2, Interferon-induced dynamin-like GTPase with antiviral activity.	**3.203**
**SOCS3**	suppressor of cytokine signaling 3, protein part of a classical negative feedback system that regulates cytokine that signal through the JAK/STAT pathway.	**1.798**

Table S2. DEGs List of the Process Network "Inflammation_Inflammasome"(Table 2); pValue: 7.669 × 10^{-3} , FDR: 5.202 × 10^{-2}, Gene Ratio: 10/119

GENE	DESCRIPTION	FC
**EIF2S3**	eIF-2-gamma X, subunit of eukaryotic initiation factor 2 (eIF2), involved in the early steps of protein synthesis.	**-1.275**
**I-kB**	Nuclear factor of kappa light polypeptide gene enhancer in B cells inhibitor, beta, inhibitor of nuclear factor kappa-light-chain-enhancer of activated B cells (NF-kappaB).	**1.475**
**IL-18**	Interleukin 18, proinflammatory cytokine primarily involved in polarized T-helper 1 (Th1) cell and natural killer (NK) cell immune responses. Upon binding to IL18R1 and IL18RAP, forms a signaling ternary complex which activates NF-kappa-B, triggering synthesis of inflammatory mediators.	**1.968**
**IRF7**	Interferon regulatory factor 7, key transcriptional regulator of type I interferon (IFN)-dependent immune responses. Regulates the transcription of type I IFN genes (IFN-alpha and IFN-beta) and IFN-stimulated genes (ISG) by binding to an interferon-stimulated response element (ISRE) in their promoters.	**3.640**
**ISG15**	ISG15 ubiquitin-like modifier, Ubiquitin-like protein which plays a key role in the innate immune response to viral infection.	**1.822**
**JNK(MAPK8-10)**	c-Jun N-terminal kinases, a group of the stress-activated protein kinases/c-Jun amino terminal kinases (SAPK/JNK). These kinases are activated by various cell stimuli and target specific transcription factors, therefore mediating gene expression in response to stimuli.	**-1.196**
**PRKRA (PACT)**	Protein activator of interferon induced protein kinase EIF2AK2 . Activates EIF2AK2/PKR in the absence of double-stranded RNA (dsRNA), leading to phosphorylation of EIF2S1/EFI2-alpha and inhibition of translation and induction of apoptosis.	**-1.162**
**RIG-I**	RIG-I-like receptor 1, innate immune receptor which acts as a cytoplasmic sensor of viral nucleic acids and plays a major role in sensing viral infection and in the activation of a cascade of antiviral responses including the induction of type I interferons and proinflammatory cytokines.	**1.700**
**TXNIP (VDUP1)**	Thioredoxin interacting protein, Thioredoxin is a thiol-oxidoreductase that is a major regulator of cellular redox signaling which protects cells from oxidative stress. This protein inhibits the antioxidative function of thioredoxin resulting in the accumulation of reactive oxygen species and cellular stress. This protein also functions as a regulator of cellular metabolism and of endoplasmic reticulum (ER) stress.	**4.596**

Table S3. DEGs List of the Process Network "signal trasduction wnt signaling" (Table 2); pValue: 6,760 × 10^{-7}, FDR: 5,307 × 10^{-5}, Gene Ratio: 21/177.

GENE	DESCRIPTION	FC
**Adenylate cyclase**	Adenylate cyclase_(MOUSE), Adenylate cyclases is a group of membrane proteins. It produces cAMP using ATP as its substrate. The group of mammalian adenylate cyclases consists of nine closely related membrane-bound forms.	**1.362**
**AKAP12**	A kinase (PRKA) anchor protein (gravin) 12, Anchoring protein that mediates the subcellular compartmentation of protein kinase A (PKA) and protein kinase C (PKC).	**-1.260**
**Casein kinase II, alpha chains**	Casein kinase II, alpha chains_(MOUSE), Group of Casein kinase II alpha catalytic subunits mediates Casein kinase II kinase activity.<br>Phosvitin/casein kinases type II are ubiquitous, highly conserved enzymes consisting of alpha, alpha-prime, and beta subunits.	**-1.242**
**FZD2**	Frizzled class receptor 2, Receptor for Wnt proteins. Most of frizzled receptors are coupled to the beta-catenin canonical signaling pathway, which leads to the activation of disheveled proteins, inhibition of GSK- 3 kinase, nuclear accumulation of beta-catenin and activation of Wnt target genes. A second signaling pathway involving PKC and calcium fluxes has been seen for some family members.	**-1.895**
**JNK2(MAPK9)**	Mitogen-activated protein kinase 9, Serine/threonine-protein kinase involved in various processes such as cell proliferation, differentiation, migration, transformation and programmed cell death. Extracellular stimuli such as proinflammatory cytokines or physical stress stimulate the stress-activated protein kinase/c-Jun N-terminal kinase (SAP/JNK) signaling pathway and a phosphorylation cascade. Promotes stressed cell apoptosis by phosphorylating key regulatory factors including TP53 and YAP1. Plays an important role in the osmotic stress-induced epithelial tight-junction disruption. When activated, promotes beta-catenin/CTNNB1 degradation and inhibits the canonical Wnt signaling pathway.	**-1.196**
**Lef-1**	Lymphoid enhancer binding factor 1, participates in the Wnt signaling pathway. Binds DNA in a sequence-specific manner and can activate transcription of target genes. Regulates T-cell receptor alpha enhancer function.	**1.451**
**MMP-2**	Matrix metallopeptidase 2, This gene encodes a member of the matrix metalloproteinase family of extracellular matrix-degrading enzymes that are involved in tissue remodeling, wound repair, progression of atherosclerosis and tumor invasion.	**1.738**
**NANOG**	Nanog homeobox, Transcription regulator involved in inner cell mass and embryonic stem (ES) cells proliferation and self-renewal. Acts as a transcriptional activator or repressor.	**-1.297**
**NF-AT2(NFATC1)**	Nuclear factor of activated T cells, cytoplasmic, calcineurin dependent 1. Proteins belonging to this family of transcription factors play a central role in inducible gene transcription during immune response.	**1.386**
**p73**	Transformation related protein 73. This gene encodes tumor protein p73, which is a member of the p53 family of transcription factors involved in cellular responses to stress and development. It can transactivate p53-responsive genes causing cell cycle arrest and apoptosis.	**-1.318**
**PP2A regulatory (Ppp2r5b)**	PP2A regulatory (MOUSE) is a trimeric complex that is composed of a catalytic C subunit, a structural A subunit, and a regulatory B subunit. Ppp2r5b particularly is the regulatory subunit B', beta, which acts as a targeting module that directs the enzyme to various intracellular locations and also provides distinct substrate specificity.	**-1.138**
**WISP1**	Cellular communication network factor 4. It is a downstream regulator in the Wnt/Frizzled-signaling pathway (By similarity). Associated with cell survival. Adheres to skin and melanoma fibroblasts (By similarity). In vitro binding to skin fibroblasts occurs through the proteoglycans, decorin and biglycan (By similarity). Suppresses tumor growth in vivo.	**-1.585**
**WNT5b**	Wingless-type MMTV integration site family, member 5B. This is a ligand for members of the frizzled family (Fzd 2). It has been proposed to be implicated in a non-canonical WNT signaling and an inhibition role in the canonical β-catenin signaling. It occurs in inflammation and IL-6 induction and CXCL8 secretion (in pulmonary fibroblasts), through JNK, p38, and p65 NF-κB activation.	**2.037**

Table S4. DEGs List of the Process Network "Cell adhesion_Cell-matrix interactions" (Table 2); pValue: 3,832 × 10^{-5}, FDR: 2,006 × 10^{-3}, Gene Ratio: 20/211

GENE	DESCRIPTION	FC
**ADAM23**	Disintegrin and metallopeptidase domain 23, Predicted to have metalloendopeptidase activity. Involved in cellular response to leukemia inhibitory factor.	**1.727**
**ADAMTS5 (Aggrecanase-2)**	ADAM metallopeptidase with thrombospondin type 1 motif 5 is a zinc-dependent aggrecanase enzyme, member of a family of multi-domain matrix-associated metalloendopeptidases that have diverse roles in tissue morphogenesis and pathophysiological remodeling, in inflammation and in vascular biology.	**-2.148**
**BETA-IG-H3 (TGFBI)**	Transforming growth factor, beta induced, exhibits extracellular matrix binding activity. Involved in cell adhesion and extracellular matrix organization. Localizes to the extracellular matrix and extracellular space.<br>Orthologous to human TGFBI (transforming growth factor beta induced).	**1.819**
**COL16A1**	This gene encodes the alpha chain of type XVI collagen, a member of the FACIT collagen family (fibril-associated collagens with interrupted helices) involved in maintaining the integrity of the extracellular matrix.	**1.402**
**COL5A1**	Collagen type V alpha 1 chain exhibits extracellular matrix structural constituent. Involved in several processes, including circulatory system development; collagen fibril organization; and tendon development. Localizes to the basement membrane and collagen trimer.	**1.355**
**Connexin 43**	Gap junction protein, alpha 1, exhibits several functions, including beta-tubulin binding activity; scaffold protein binding activity; and transmembrane transporter activity. Involved in several processes, including animal organ development; regulation of gene expression; and regulation of membrane depolarization. Localizes to several cellular components, including the fascia adherens; gap junction; and lateral plasma membrane. Colocalizes with the cell-cell junction.	**-1.128**
**Decorin**	Decorin, exhibits extracellular matrix binding activity and glycosaminoglycan binding activity. Involved in peptide cross-linking via chondroitin 4-sulfate glycosaminoglycan. Localizes to the collagen-containing extracellular matrix and extracellular space. According to Metacore it can inhibit TGF-B ligands by binding.	**2.915**
**ECM1**	Extracellular matrix protein 1, exhibits interleukin-2 receptor binding activity. Involved in several processes, including chondrocyte development; regulation of T cell migration; and regulation of bone mineralization. Localizes to the extracellular space.	**1.401**
**EMILIN-2**	Elastin microfibril interfacer 2, predicted to have extracellular matrix constituent conferring elasticity. Predicted to be involved in cell adhesion. Localizes to the collagen-containing extracellular matrix and extracellular space. Is expressed in embryo mesenchyme.	**1.771**
**ITGB7**	Integrin beta 7, predicted to have integrin binding activity. Involved in T cell migration. Predicted to localize to the cell surface; focal adhesion; and integrin alpha4-beta7 complex. It is expressed in genitourinary system and thymus primordium.	**1.503**
**LAMA4**	Laminin, alpha 4, predicted to have extracellular matrix structural constituent and signaling receptor binding activity. Involved in blood vessel development; brown fat cell differentiation.	**1.681**
**MMP-12**	Matrix metallopeptidase 12, predicted to have several functions, including DNA binding activity; metal ion binding activity; and metalloendopeptidase activity. Involved in several processes, including positive regulation of interferon-alpha secretion; regulation of gene expression; and regulation of type I interferon-mediated signaling pathway. Localizes to the extracellular space and nucleus.	**-5.146**
**MMP-13**	Matrix metallopeptidase 13, exhibits metalloendopeptidase activity. It is involved in several processes, including bone mineralization; collagen catabolic process; and endochondral bone morphogenesis. Predicted to localize to several cellular components, including the Golgi apparatus; intercellular canaliculus; and lysosome. Its expression can be stimulated by interferon-b action and IL-18 62	**1.793**
**MMP-2**	Matrix metallopeptidase 2, exhibits metalloendopeptidase activity. Involved in several processes, including blood vessel maturation; bone trabecula formation; and collagen catabolic process. Localizes to several cellular components, including the extracellular space; mitochondrion; and sarcomere.<br>It is involved in the regulation of IL6/Stat3 survival signaling 62	**1.738**
**Perlecan**	Perlecan (heparan sulfate proteoglycan 2), exhibits protease binding activity. Involved in several processes, including animal organ development; extracellular matrix organization; and skeletal system development. Localizes to the basement membrane.	**1.137**

**TSG-6**	Tumor necrosis factor alpha induced protein 6, predicted to have hyaluronic acid binding activity. Predicted to be involved in negative regulation of inflammatory response and positive regulation of cell migration. Predicted to localize to the extracellular space.	**1.603**
**Versican**	Versican, exhibits protein phosphatase binding activity. Involved in development (embryo mesenchyme).	**1.371**
**WISP1**	Cellular communication network factor 4, predicted to have heparin binding activity and integrin binding activity. Involved in several processes, including glucose homeostasis; negative regulation of cell differentiation; and osteoclast differentiation. Localizes to the cytoplasm.	**-1.585**
